# Major congenital anomalies in children born to women with systemic lupus erythematosus

**DOI:** 10.1186/ar3945

**Published:** 2012-09-27

**Authors:** E Vinet, CA Pineau, AE Clarke, M Kaouache, C Gordon, R Platt, S Bernatsky

**Affiliations:** 1 McGill University Health Centre, Montreal, QC, Canada; 2 McGill University, Montreal, QC, Canada; 3 Rheumatology Research Group, University of Birmingham, Birmingham, UK

## Background

Systemic lupus erythematosus (SLE) can cause considerable morbidity during pregnancy. Although several studies have evaluated fetal outcome in lupus pregnancy, only one small study assessed congenital anomalies in 30 children born to SLE mothers, showing no excess risk. In a large population-based study, we aimed to determine whether children born to women with SLE have an increased risk of major congenital anomalies compared with children born to women without SLE.

## Methods

We identified all women who had ≥1 hospitalization for a delivery after SLE diagnosis using Quebec's physician billing and hospitalization databases (1989 to 2009). Women were defined as SLE cases if they had any of the following: ≥1 hospitalization with a diagnosis of SLE prior to the delivery; a diagnosis of SLE recorded at the time of their hospitalization for delivery; or ≥2 physician visits with a diagnosis of SLE, occurring 2 to 24 months apart, prior to the delivery. We randomly selected a general population control group, composed of women matched at least 4:1 for age and year of delivery, who did not have a diagnosis of SLE prior to or at the time of delivery. We identified children born live to SLE cases and their matched controls, and obtained information on all physician visits and hospitalizations incurred by these children. We ascertained major congenital anomalies, as defined by ≥1 hospitalization or physician visit with an ICD-9/10 diagnostic code for major congenital anomaly, within the first 12 months of life. We performed multivariate analyses to adjust for maternal demographics (that is, age, education, marital status), sex and birth order of child, major maternal co-morbidities (that is, pre-gestational diabetes, hypertension, asthma, depression), and relevant maternal medications (that is, antimalarials, corticosteroids, immunosuppressants).

## Results

A total of 507 women with SLE had 721 children, while 5,862 matched controls had 8,561 children. Compared with controls, children born to women with SLE experienced slightly more major congenital anomalies (13.6% (95% CI = 11.3 to 16.3) vs. 10.4% (95% CI = 9.7 to 11.1)). In multivariate analyses, children born to women with SLE had a substantially increased risk of major congenital anomalies (adjusted OR = 1.28, 95% CI = 1.01 to 1.62) compared with controls. Medication exposures did not seem to mediate the risk of major congenital anomalies.

## Conclusion

Our findings suggest that, compared with children from the general population, children born to women with SLE have an increased risk of major congenital anomalies, and prompt further research to elucidate this issue.

